# Beneficial effects of *Cichorium intybus L.* extract on oxidative status and reproductive parameters in male Wistar rats: An experimental study

**DOI:** 10.18502/ijrm.v17i6.4814

**Published:** 2019-07-29

**Authors:** Mehran Dorostghoal, Seyyed Mansour Seyyednejad, Marzieh Noroozi Tabrizi Nejad

**Affiliations:** Department of Biology, Faculty of Science, Shahid Chamran University of Ahvaz, Ahvaz, Iran

**Keywords:** Spermatogenesis, Oxidativestress, Rat, Cichorium intybus L.

## Abstract

**Background:**

During recent years, increasing concern has been raised about the declining sperm count and human male infertility. *Cichorium intybus* L. (*C*. *intybus*) has traditionally been used in Iranian folk medicine as hepato protective and blood purifier and for its presumed fertility-enhancing properties.

**Objective:**

A dose-response study was performed to determine the effect of *C*. *intybus* ethanolic leave extract on the reproductive parameters in adult Wistar male rats.

**Materials and Methods:**

In this experimental study, 40 healthy adult male Wistar rats (8 wk old, 200-210 gr body weight) were randomly divided (n = 10/each) as control and groups treated with 50, 100, and 200 mg/kg/day of *C. intybus* extract via gavage for 70 days. Serum hormonal assay, epididymal sperm evaluation, and analysis of morphometrical parameters, antioxidant enzymes, and lipid peroxidation levels of testis were done in each experimental group.

**Results:**

Weights of testis and epididymis increase significantly in male rats treated with 200 mg/kg *C. intybus* extract. Sperm density and percent of morphologically normal sperm were significantly increased in a dose-related manner with *C. intybus* treatment.Serum testosterone was higher at 100 and 200 mg/kg *C. intybus* extract-treated groups.* C. intybus* significantly reduced malondialdehyde levels and also increased superoxide dismutase and glutathione peroxidase activity in testicular tissue of rats.

**Conclusion:**

It is concluded that *C. intybus* leave extract improves reproductive parameters in male rats which might be a consequence of both its antioxidant and androgenic properties.

## 1. Introduction

In recent years, concerns about the potential detrimental effects of occupational and environmental exposures to heat, radiation, and heavy metals and endocrine-disrupting chemicals in deteriorating sperm quality and affecting male fertility have been raised (1). In this regard, it is suggested that excess production of free radicals and oxidative stress have been associated in the pathophysiology of male reproduction (2). Because sperm cell membrane is rich in polyunsaturated fatty acids and phospholipids, it is highly susceptible to a high concentration of free radicals and oxidative damage have been suggested as one of the possible etiologies of idiopathic male infertility (3). However, it is suggested that the treatment with antioxidant compounds might have beneficial effects on male infertility (4). Traditional plants are used as a source of treatment of diseases in different parts of the world, of which including antioxidant rich plants because of their health-promoting properties. It is thought that supplementation with certain plants could reduce the toxicity of some toxicants including occupational and environmental agents (5).

*Cichorium intybus* L. (*C. intybus*) (Asteraceae) is an herbaceous plant that grows in different area of Europe, Africa, and Asia. *C. intybus* is an erect, glandular, biennial plant with a tuberous taproot and rosette of 30-70 leaves. In Europe *C. intybus* is grown as a leaf vegetable or salad green and particularly in India and South Africa its roots are often used as a coffee substitute or supplement (6). *C. intybus* has been found to have many usage in the food industry and has been used traditionally in Ayurvedic and Unani systems to cure different diseases (7).

*C. intybus* is known as Kasni in Iran and its seeds and leaves have traditionally been considered in Iranian folk medicine to be hepatoprotective and blood purifier (8, 9), and also to have male fertility-promoting properties (10, 11). The whole extract of *C. intybus* was reported to have anti-diabetic (12), antibacterial (13), immunotoxic (14), and cardioprotective properties (15). Inulin, fructooligosaccharides, polyphenols such as chlorogenic acid and caffeic acid derivatives are the phytochemical components of *C. intybus* (16). Chicory leaves are rich of natural antioxidants for pharmaceutical or dietary needs. The aqueous and alcoholic extracts of chicory showed significant protection against protein oxidation and DNA damage due to presence of phenolic compounds that posses marked radical scavenging properties (7, 17). However, effects of *C. intybus* extract on the male reproductive organs and functions have not been studied.

Therefore, this work was done to evaluate the effects of different doses of *C. intybus* ethanolic leave extract on the testicular oxidative status and reproductive parameters in adult Wistar male rats.

## 2. Materials and Methods

### Plant materials

*C. intybus* leaves were collected from Behbahan city in Khouzestan province of Iran in March 2012. It was recognized and authenticated in Botanical Systematic Laboratory, Department of Biology, Shahid Chamran University of Ahvaz, Iran.

### Preparation of ethanolic extract of C. intybus

*C. intybus* fresh leaves of were dried at 40°C for 48 hr, powdered in an electrical grinder and extracted successively with ethanol. The mixture was filtered after extraction and the solvent evaporated to dryness the extract left behind (yield was 5.8 g/kg) stored at 4°C and dissolved in distilled water whenever needed for experiments (18).

### Chemicals

All chemicals were purchased from Merck, Darmstadt, Germany. Testosterone assay kit (Monobind Inc., California, USA), luteinizing hormone and follicle-stimulating hormone kits (AccuBind ELISA Kits, California, USA) and Glutathione peroxidase (GPx) and superoxide dismutase (SOD) kits (Randox Labs Ltd. Ardmore, United Kingdom) were used for hormonal and biochemical assays.

### Animals

In this experimental study, 40 healthy adult male Wistar rats (8 wk old, 200-210 gr body weight) were housed under standard laboratory conditions (temperature 23 ± 2°C, relative humidity of 50% ± 5%, 12hr light/dark cycle) and were given food and water ad libitum. The animals were obtained from the Research Center and Experimental Animal House of Ahwaz Jundishapour, University of Medical Sciences.

### Experimental design

Animals after one week of acclimatization were divided randomly into four equal groups each contain ten *rats. In *control group, rats were administered with 1 ml distilled water via gavage once daily for 70 days. In treatment groups, rats were administered with 50, 100 and 200 mg/kg body weight ethanolic extract of *C. intybus* via gavage once daily for 70 days. In order to evaluate *C. intybus* effect through a complete spermatogenic cycle, male rats were exposed to its extract for 70 days (19).

At the termination of the experiment the body weight of rats was recorded. The animals were sacrificed under light ether anesthesia, and then blood samples were collected by heart puncture, the reproductive organs were removed and weighed. Left testes were minced and homogenized for biochemical assays and right testes were immediately fixed into Bouin's solution for morphometrical analysis.

### Morphometrical analysis

Serial paraffin embedding sections (5 µ thickness) were stained with haematoxylin and eosin and mean of two perpendicular diameters of each seminiferous tubules cross-section and germinal epithelium height in four equidistance of each seminiferous tubules cross-section were measured using an ocular micrometer of light microscopy (Olympus BH, Japan, Tokyo) (20).

### Sperm analysis 

The epididymal tail was trimmed in 1.0 ml of 0.1 M phosphate buffer of pH 7.4, an aliquot of 10 μL was placed in a hemocytometer chamber (Paulmarine, Germany) and one hundred sperm were evaluated per animal and classified into motile and immotile. 10 μL aliquot of the epididymal sperm suspension was transferred to each hemocytometer counting chamber for the evaluation of sperm density. Eosin staining method was used for analysis of sperm morphology. Two hundred sperm per animal were examined and the number of morphologically abnormal sperm was recorded (21).

### Tissue biochemistry

The left testes dissected free from the surrounding fat and homogenized (10% w/v) in ice-cold 0.1 M sodium phosphate buffer. The testicular homogenates were centrifuged (10000 rpm for 15-20 min at 4°C) and glutathione peroxidase levels and superoxide dismutase activity were analyzed in the resulting supernatant using colorimetric assay kits (Randox Labs Ltd., Ardmore, United Kingdom). The amount of malondialdehyde (MDA) in the testis were assayed using the thiobarbituric acid test as described by Ohkawa and co-workers (22).

### Serum hormonal assay

Blood samples were centrifuged (10 min at 2430 × g), and testosterone, luteinizing hormone and follicle-stimulating hormone levels in serum were measured by radioimmunoassay using the commercial kit (AccuBind ELISA Kits, California, USA) according to the kit manufacturer's instructions.

### Ethical consideration

The animals were handled according to the guidelines of the National Research Council Guide for the Care and Use of Laboratory Animals. The Animal Ethics Committee of Shahid Chamran University of Ahvaz has approved the experimental protocol.

### Statistical analysis

The data were analyzed using the analysis of variance (ANOVA) followed by Tukey's test (SPSS, version 16, SPSS Inc., Chicago, Illinois, USA). The level of significance was set at p < 0.05 and data are expressed as the Mean ± SEM.

## 3. Results

### Clinical signs of toxicity

Oral administration of *C. intybus* extract did not produce any visible signs of toxicity in any of the experimental rats.

### Body and reproductive organs weight

The treatment of rats with *C. intybus* extract caused no difference on the body weight as compared with the control group. However, significant (p < 0.001) increases were seen in the testis weight of male rats treated with 200 mg/kg *C. intybus* extract in comparison with the control group. Also, epididymis weight was statistically higher in rats treated with 100 (p < 0.001) and 200 mg/kg (p < 0.001) *C. intybus* extract. There were no statistically significant differences in the weights of ventral prostate and seminal vesicle between extract-treated and control groups (Table I).

### Morphometrical analysis

**Figure 1:**
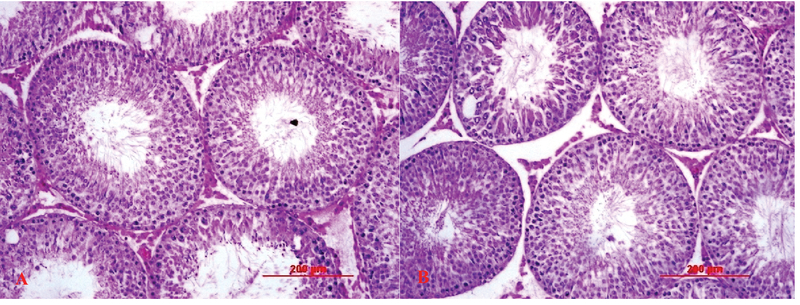
Histological sections of Wistar rats testis in experiment groups; control (A) and 200 mg/kg/day *C*. *intybus* extract-treated group (B). GEH: Germinal Epithelium Height; INT: Interstitium; LUM: Lumen, 40×, H&E.

Seminiferous tubules diameter and germinal epithelium height were significantly higher (p<0.001) in male rats treated with 100 and 200 mg/kg *C. intybus* extract in comparison with control rats (Figure 1; Table II).

### Sperm analysis

Significant dose-related increases in sperm density observed after administration of *C. intybus *to adult male rats. Also, the percentage of morphologically abnormal sperm was statistically reduced in rats treated with 100 (p = 0.005) and 200 (p = 0.001) mg/kg *C*. *intybus* extract in comparison with the control group. No significant differences were observed in the percentage of motile sperms between *C*. *intybus* extract-treated and control groups (Table II).

### Tissue biochemistry

Testis MDA levels showed significant (p < 0.001) decrease in rats treated *C*. *intybus* extract when compared with the control group. Moreover, significant increase observed in activity levels of SOD and GPx (p < 0.001) in the testis of *C*. *intybus* extract-treated rats as compared to the control group (Table III).

### Serum hormonal assay

**Figure 2:**
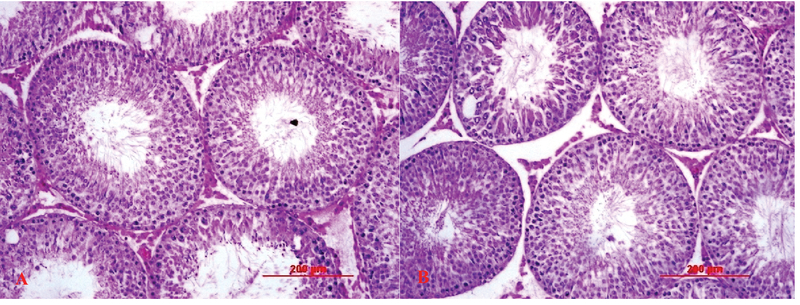
Effects of *C. intybus* extract on serum testosterone, LH, and FSH levels in adult male Wistar rats. ${}^{*}$: Differ significantly (p < 0.05) with respect to control rats. a, b, and c: indicates significant (p < 0.05) differences between treatment groups. ANOVA followed by Tukey's test. Data are presented as mean ± SEM LH: Luteinizing Hormone; FSH: Follicle-Stimulating Hormone

Significant increase was seen in testosterone levels in rats treated with 100 (p = 0.01) and 200 (p < 0.001) mg/kg *C*. *intybus *extract as compared to the control group. No significant differences were observed in FSH and LH levels between *C*. *intybus* extract-treated and control groups (Figure 2).

**Table 1:** Effects of *C*. *intybus* extract on body and reproductive organs weight in Wistar rats


**Parameters**	**Control**	**** ***C. intybus*** ** (50 mg/kg) (a)**	**** ***C*** **. ** ***intybus*** ** (100 mg/kg) (b)**	**** ***C*** **. ** ***intybus*** ** (200 mg/kg) (c)**	**P-value**
Body (gr)	303.5 ± 2.7	308.3 ± 3.1	306.5 ± 3.3	309.4 ± 2.7	0.546
Testis (mg)	1341.3 ± 3.1	1348.0 ± 3.5	1355.1 ± 3.8	1409.6 ± 4.4${}^{*a}$	0.000
Epididymis (mg)	443.1 ± 3.0	457.0 ± 3.7	491.8 ± 4.2${}^{*}$ **${}^{a}$**	537.0 ± 4.3${}^{*a,b}$	0.000
Seminal vesicle (mg)	457.1 ± 4.6	453.1 ± 4.5	464.9 ± 5.2	471.1 ± 6.1	0.083
Ventral prostate (mg)	196.2 ± 1.9	203.1 ± 2.7	201.3 ± 2.5	205.3 ± 3.0	0.104
Data are presented as mean ± SEM. ANOVA followed by Tukey's test					
*: Differ significantly (p < 0.05) respect to control rats. a, b, and c: indicates significant (p < 0.05) differences between treatment groups					

**Table 2:** Effects of *C*. *intybus* extract on seminiferous tubule and sperm parameters in Wistar rats


**Parameters**	**Control**	**** ***C*** **. ** ***intybus*** ** (50mg/kg) (a)**	**** ***C*** **. ** ***intybus*** ** (100mg/kg) (b)**	**** ***C*** **. ** ***intybus*** ** (200mg/kg) (c)**	**P-value**
STD (μm)	251.7 ± 2.2	260.1 ± 2.4	268.3 ± 2.3${}^{*}$	271.0 ± 2.3${}^{*a}$	0.000
GEH (μm)	65.3 ± 1.5	71.2 ± 1.5	76.5 ± 1.6${}^{*}$	77.9 ± 1.8${}^{*a}$	0.000
Sperm count (million/ml)	60.1 ± 1.3	64.6 ± 1.5	71. 5 ± 1.6${}^{*a}$	73.0 ± 1.9${}^{*a}$	0.000
Motile sperm (%)	75.6 ± 1.6	75.1 ± 1.4	78.9 ± 1.4	79.5 ± 1.7	0.128
Abnormal sperm (%)	15.1 ± 1.0	13.4 ± 0.77	11.1 ± 0.67${}^{*}$	10.3 ± 0.61${}^{*a}$	0.000
Data are presented as mean ± SEM. ANOVA followed by Tukey's test					
*: Differ significantly (p < 0.05) with respect to control rats. a, b, and c: indicates significant (p < 0.05) differences between treatment groups					
STD: Seminiferous tubule diameter; GEH: Germinal epithelium height					

**Table 3:** Effects of *C. intybus* extract on lipid peroxidation and antioxidant enzymes levels in adult male Wistar rats


**Parameters**	**Control**	**** ***C. intybus*** ** (50mg/kg) (a)**	**** ***C*** **. ** ***intybus*** ** (100mg/kg) (b)**	**** ***C*** **. ** ***intybus*** ** (200mg/kg) (c)**	**P-value**
MDA (nmol/mg protein)	10.13 ± 1.2	7.15 ± 0.99${}^{*}$	4.65 ± 0.77${}^{*}$	2.74 ± 0.56${}^{*a}$	0.000
GPx(U/mg protein)	2.27 ± 0.29	4.30 ± 0.42${}^{*}$	6.04 ± 0.36${}^{*a}$	7.20 ± 0.37${}^{*a}$	0.000
SOD (U/mg protein)	15.30 ± 1.01	23.09 ± 1.41${}^{*}$	28.85 ± 1.57${}^{*a}$	31.30 ± 1.55${}^{*a}$	0.000
Data are presented as mean ± SEM. ANOVA followed by Tukey's test					
*: Differ significantly (p < 0.05) with respect to control rats. a, b, and c: indicates significant (p < 0.05) differences between treatment groups					
MDA: Malondialdehyde; GPx: Glutathione peroxidase; SOD: Superoxide dismutase					

## 4. Discussion

In this study, *C. intybus* leave extract effects on the reproductive and oxidative status parameters were evaluated in adult Wistar male rats. Our findings indicated that *C. intybus* leave extract has beneficial effects on reproductive functions in adult male rats. Our findings demonstrated that *C. intybus* leave extract increases testis and epididymis weights, percentage of sperm with normal morphology, sperm counts, levels of serum testosterone, SOD and GPx levels, and also decreases the MDA levels in adult male rats.

The mass of differentiated spermatogenic cells determine the weight of testis (23) and the number of stored sperm in the epididymis characterizes the weight of epididymis. Androgens play an important role in the development, growth, and normal functioning of the reproductive organs, so that the structural and functional integrity of testes, and male accessory reproductive glands depends on the adequate bioavailability of testosterone (24). The increased androgen biosynthesis as evidenced by a significant increase in serum testosterone levels in the extract-treated rats could explain the significant increase in the weight of reproductive organs. Morphometrical studies show that seminiferous tubules form the main portion of testicular tissue (20). Therefore, an increase in the testicular weight can result from the increase in the height of germinal epithelium and the diameter of seminiferous tubules seen in *C. intybus* extract-treated rats. Furthermore, our findings show that sperm count increases in a dose-related manner in *C. intybus* extract-treated rats. Epididymal sperm count is known as one of the most sensitive tests for evaluating spermatogenesis (25), and often used as a benchmark of sperm production, testicular function, and/or male fertility. High percentage of sperm with abnormal morphology and low sperm concentration each have been related to reduced fertility (26). Treatment with *C. intybus* via increase in sperm count and percentage of normal morphological sperm improves and enhances the fertilizing capacity of semen.

Improved testicular/sperm function in the present study may be caused by the increase in SOD and GPx levels and decrease in the levels of MDA in the testis of adult rats. This study showed that *C. intybus* reduces the levels of lipid peroxidation and increases the activities of SOD and GPx in testes and hereby improves the testicular oxidative status. An Increased level of MDA followed by reactive oxygen species (ROS) production potentially has harmful effects on sperm quality and function (27). Elevation of lipid peroxidation in the sperm plasma membrane, due to increase in free radicals generation and consequent oxidative stress, is known to play a critical role in the etiology of abnormal sperm function(28). Oxidative stress due to the generation of ROS leads to poor sperm quality and male infertility (29). Accordingly, sperm motility and normal morphology affect by elevation of membrane lipid peroxidation (30). Association between low sperm motility and increased levels of ROS has been reported by Agarwal and co-workers (31). So, it seems that increasing reproductive efficiency due to the improvement of the testicular oxidative status, which in present study revealed as improvement of some of the reproductive parameters, is not unexpected. In this regards, Tiya and colleagues reported that chronic treatment with *Hypoxis hemerocallidea* may prevent testicular oxidative damage and improve reproductive parameters and male fertility (32). Alabi and co-worker recommended *Nauclea latifolia* as a natural product for treatment of male infertility. Improvement in testicular oxidative status, androgenicity, and sperm quality, following administration of *Nauclea latifolia* has been reported (33).

Anti-free radical activity of *C. intybus* constituents, especially from the red varieties, has been shown by in-vitro studies (34). Jamshidzadeh and colleagues showed that the *C. intybus* extract could protect the liver from CCl4-induced oxidative stress (18). Gadgoli and Mishra (35) also showed that the levels of alanine aminotransferase (ALT) and aspartate aminotransferase (AST) reduced in paracetamol-induced hepatotoxicity by total aqueous and alcoholic extract of *C. intybus* seeds. Moreover, phytochemical analyses showed that *C. intybus* alcoholic extract contains high amounts of phenolic compounds (7). Antioxidant properties of phenolic compounds for protecting against cellular oxidative damage due to their hydrogen-donating ability have been studied extensively. Phenolic compounds, by decreasing membrane fluidity in a concentration-dependent manner prevent the diffusion of free radicals and restrict peroxidative reaction, stabilize membranes (36). However, it has been reported that radical scavenging properties of chicory preparations is related to not only phenolic compounds but also sugars, especially sucrose and fructans (37).

## 5. Conclusion

Improvement of spermatogenesis and male reproductive parameters observed after administration of *C. intybus* leave extract, which might be a consequence of both its potent antioxidant properties and androgenic activities. Our findings support the traditional use of *C. intybus* as a solution for male reproductive problems. However, the toxicity and therapeutic efficacy of various fractions of *C. intybus* needs to be investigated in future studies.

##  Conflict of Interest

The authors declare that there are no conflicts of interest.
